# 4-Pyridoxic Acid in the Spent Dialysate: Contribution to Fluorescence and Optical Monitoring

**DOI:** 10.1371/journal.pone.0162346

**Published:** 2016-09-06

**Authors:** Sigrid Kalle, Risto Tanner, Jürgen Arund, Ruth Tomson, Merike Luman, Ivo Fridolin

**Affiliations:** 1 Department of Biomedical Engineering, Technomedicum, Tallinn University of Technology, Tallinn, Estonia; 2 Centre of Nephrology, North Estonian Medical Centre, Tallinn, Estonia; Hospital Universitario de la Princesa, SPAIN

## Abstract

**Aim:**

In this work we estimated the contribution of the fluorescence of 4-pyridoxic acid (4-PA) to the total fluorescence of spent dialysate with the aim of evaluating the on-line monitoring of removal of this vitamin B-6 metabolite from the blood of patients with end-stage renal disease (ESRD).

**Methods:**

Spectrofluorometric analysis of spent dialysate, collected from hemodialysis and hemodiafiltration sessions of 10 patients receiving regularly pyridoxine injections after dialysis treatment, was performed in the range of Ex/Em 220–500 nm. 4-PA in dialysate samples was identified and quantified using HPLC with fluorescent and MS/MS detection.

**Results:**

Averaged HPLC chromatogram of spent dialysate had many peaks in the wavelength region of Ex320/Em430 nm where 4-PA was the highest peak with contribution of 42.2±17.0% at the beginning and 47.7±18.0% in the end of the dialysis. High correlation (R = 0.88–0.95) between 4-PA concentration and fluorescence intensity of spent dialysate was found in the region of Ex310-330/Em415-500 nm, respectively.

**Conclusion:**

4-PA elimination from the blood of ESRD patients can be potentially followed using monitoring of the fluorescence of the spent dialysate during dialysis treatments.

## Introduction

Vitamin deficiency is common in chronic kidney disease (CKD) patients. One vitamin that CKD patients are lacking is vitamin B-6 (B6) which is the term for a group of interconvertible molecules containing pyridoxine, pyridoxal, pyridoxamine and their phosphates. The deficiency of B6 has been linked to many pathologies including impaired gluconeogenesis and glucose tolerance [1], metabolism of amino acids [2], regulation of the level of circulating insulin [3,4], diabetes type 1 [5] and type 2 [6]. Many studies have pointed out that lower vitamin B-6 concentrations increase the risk of coronary artery disease [7–9] as well as renal dysfunction [10]. Concerning uremia it is essential to consider metabolic link between urea accumulation in uremic tissues and inactivation of pyridoxal-5’-phosphate (PLP) by carbamoyl phosphate, the very first product of condensation of carbonate with ammonia in the metabolic pathway of urea biosynthesis [11] leading to symptoms of B6 deficiency [12]. The deficit of B6 in dialysis patients is treated by regular B6 administration [13–15] but there is no adequate consideration of potential toxicity of larger doses. Levine *et al*. have found that decreased excretion of pyridoxine and its metabolites might increase the likelihood to pyridoxine toxicity [16] and thus status of pyridoxine monitoring would be beneficial.

The main active form of B6 is pyridoxal-5’-phosphate (PLP). PLP acts as a cofactor for 147 EC-classified enzymes, 64 of which are known to be present in multicellular animals [17]. PLP has been widely employed as a status indicator of vitamin B-6 but recently usage of other vitamers and 4-pyridoxic acid has been proposed [18]. 4-PA is the major urinary catabolite of B6 [19] since it has been estimated that in healthy individuals 40–60% of ingested B6 is oxidized to 4-pyridoxic acid [7,20]. 4-PA can be measured in blood plasma or serum with high sensitivity using HPLC with different modifications such as post-column derivatization [21,22] etc. These methods require blood sampling from the patient and several laborious steps, which are expensive and time consuming.

Our previous studies have shown that UV and fluorescence spectra data, measured directly at the outflow of the spent dialysate from dialysis machine, can be used to calculate contents of different characteristic uremic solutes [23,24]. The benefit of such information is the possibility of on-line monitoring of the dialysis process and rapid correction of the treatment depending on the status of the patient being treated.

The aim of this study was to investigate the potential of measurement of fluorescence in spent dialysate for monitoring of the elimination of 4-PA from the blood of dialysis patients receiving regular B6 treatment. The set aim was achieved.

## Materials and Methods

### 2.1. Ethics

The study was approved by the Tallinn Medical Research Ethics Committee at the National Institute for Health Development, Estonia decision no. 2349. A written informed consent was obtained from all participating patients.

### 2.2. Patients and samples

39 dialysis sessions of 10 patients (age 59 ± 15 years) were followed. 100 mg B6 was routinely injected to patients after each dialysis session. The dialysis machine used was Fresenius 5008H (Fresenius Medical Care, Germany), dialyzers were FX8 or FX1000, the dialysate and blood flow varied from 500–800 mL/min and 300–350 mL/min, respectively. The dialysate samples were collected 7–10, 60, 120, 180, 240 minutes after the start of the dialysis session from the outlet dialysate line and from tank (145 HD and 50 HDF samples in total). All dialysate samples were acidified down to pH 4.25 with formic acid before the HPLC analysis for the best chromatographic separation and stable retention times. Full fluorescence spectra of spent dialysates in the range of excitation/emission 220–500 nm and emission with excitation increment 10 nm were recorded with the spectrofluorophotometer RF-5301 by Shimadzu (Kyoto, Japan). The cell with optical path 4 mm was used for measurement and the Panorama Fluorescence 1.2 software by Shimadzu for spectral data processing.

### 2.3. HPLC and MS system

The HPLC system consisted of a gradient pump unit, a thermostated auto sampler, a column oven, a diode array spectrophotometric detector (DAD) and a fluorescence detector (FLD), all Ultimate 3000 Series instruments from Dionex (Sunnyvale, CA, USA), column of Kinetex C18 100A column (Phenomenex, USA) with a security guard KJO-4282 from Phenomenex (Torrance, CA, USA). The fluorescence was recorded at the wavelength of Ex320/Em430 nm and measurement interval of 0.5 s. Chromatographic data was processed with Chromeleon 7.1 software by Dionex Thermo Scientific (Waltham, USA).

The micrOTOF-Q II instrument by Bruker Daltonik GmbH (Bremen, Germany) with ESI source was used for mass-spectrometric analyses. For the identification of 4-PA both positive and negative ion mode were used. Sample analysis were done with the following parameters: mass range of 60–1700 m/z, ion source temperature of 200°C, ESI voltage of 4.5 kV, ESI nebulization gas flow of 8.0 L/min, drying gas flow of 1.2 bar, detector voltage of 2.03 kV and acquisition rate of 1 Hz. Mass calibration was performed with sodium formate solutions from m/z 60 to 1700. For data acquisition software Compass HyStar version 3.2 and for processing Compass DataAnalysis version 4.0 SP1 was used (both Bruker, Billerica, USA).

### 2.4. Mobile phase

The two-component eluent was used as mixture of A: 0.05 M formic acid adjusted to pH 4.25 with ammonium hydroxide and B: the mixture of methanol and acetonitrile in the volume ratio of 9:1, both HPLC-grade from Rathburn (Walkerburn, Scotland). The five-step linear gradient elution program was used, as specified in Table 1.

**Table 1:** HPLC gradient program.

Step	Time (min)	Buffer (A) %	Organic solvent (B) %	Curve type
0	0	100	0	
1	0	100	0	linear
2	30	90	10	linear
3	60	5	95	concave
4	80	5	95	linear
5	82	100	0	linear

The total flow rate of 0.8 mL/min was used with the column temperature of 40°C. The sample volume injected was 20–50 μL.

### 2.5. Identification and contribution

Chromatographic peak of the 4-PA was identified by comparing retention time, UV absorption, fluorescence and mass spectra data of an unknown found in the sample with the corresponding characteristics of the reference compound (4-pyridoxic acid, Sigma Aldrich, USA). HPLC fluorescence data of reference 4-PA solution with different known concentrations were used to create a calibration curve. Concentration of 4-PA was calculated on the basis of HPLC fluorescence chromatograms.

The relative contribution (RC) of the 4-PA peak in the total fluorescence of the samples was calculated as a ratio of the area of 4-PA peak (A_PA_) to the total area of all peaks appeared on the chromatogram (A_total_): RC (%) = (A_PA_/A_total_)*100

Student’s t-test was used to compare Two-Sample dataset, Assuming Unequal Variances, while p < 0.05 was considered significant.

## Results

### 3.1. Emission spectra and average HPLC fluorescence chromatogram of spent dialysate

**Fig 1:**
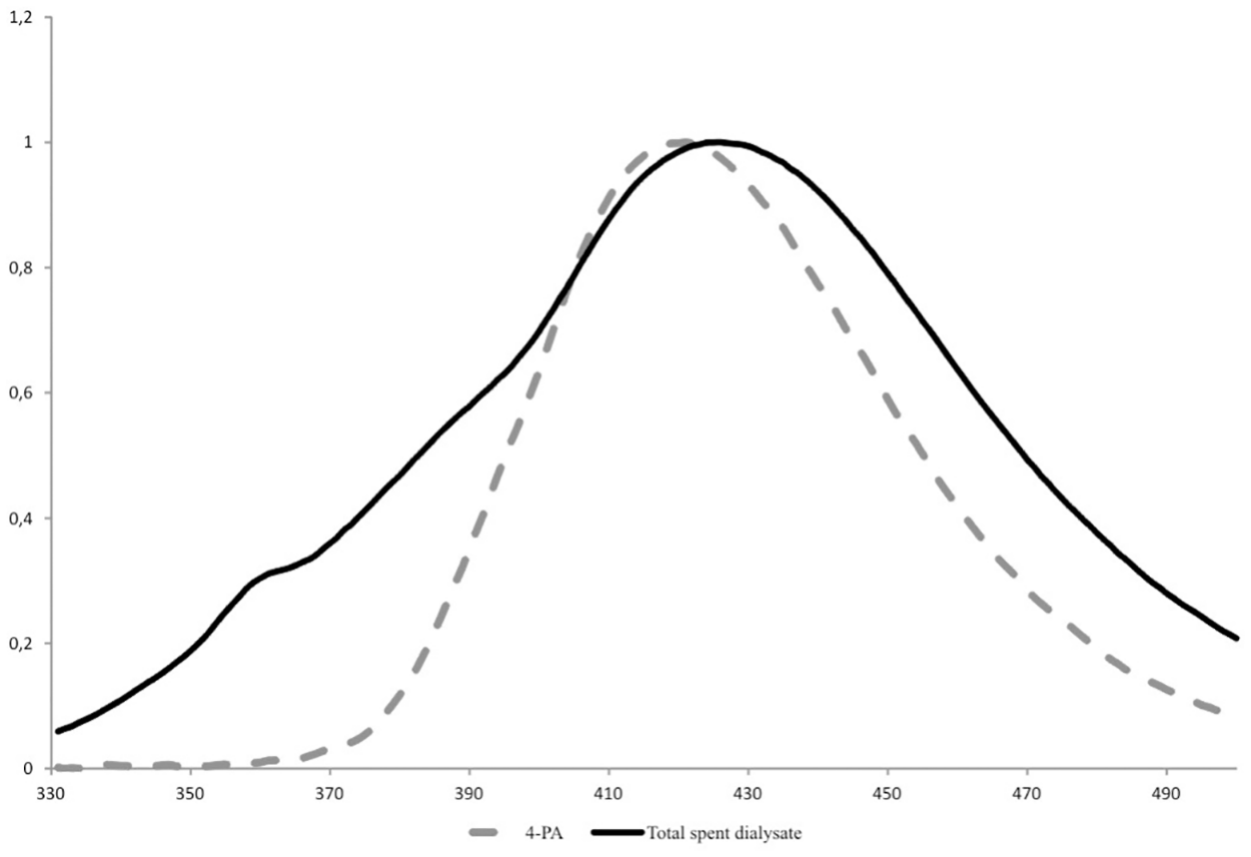
Normalized emission spectra of spent dialysate (solid line) and 4-pyridoxic acid (dotted line) in NaHCO_3_/acetate buffer pH 7,6. Excitation at 320 nm.

Fig 1 presents the characteristic emission spectrum of spent dialysate compared with the reference spectrum of the 4-PA in the same buffer solution (sodiumbicarbonate/acetate pH 7.6; Fresenius Medical Care AG &Co) (for specific data see Figure A in S1 file). The graph presents quite similar Em spectra shape in the wavelength region 400–500 nm for the total fluorescence signal of spent dialysate and fluorescence of 4-PA.

**Fig 2:**
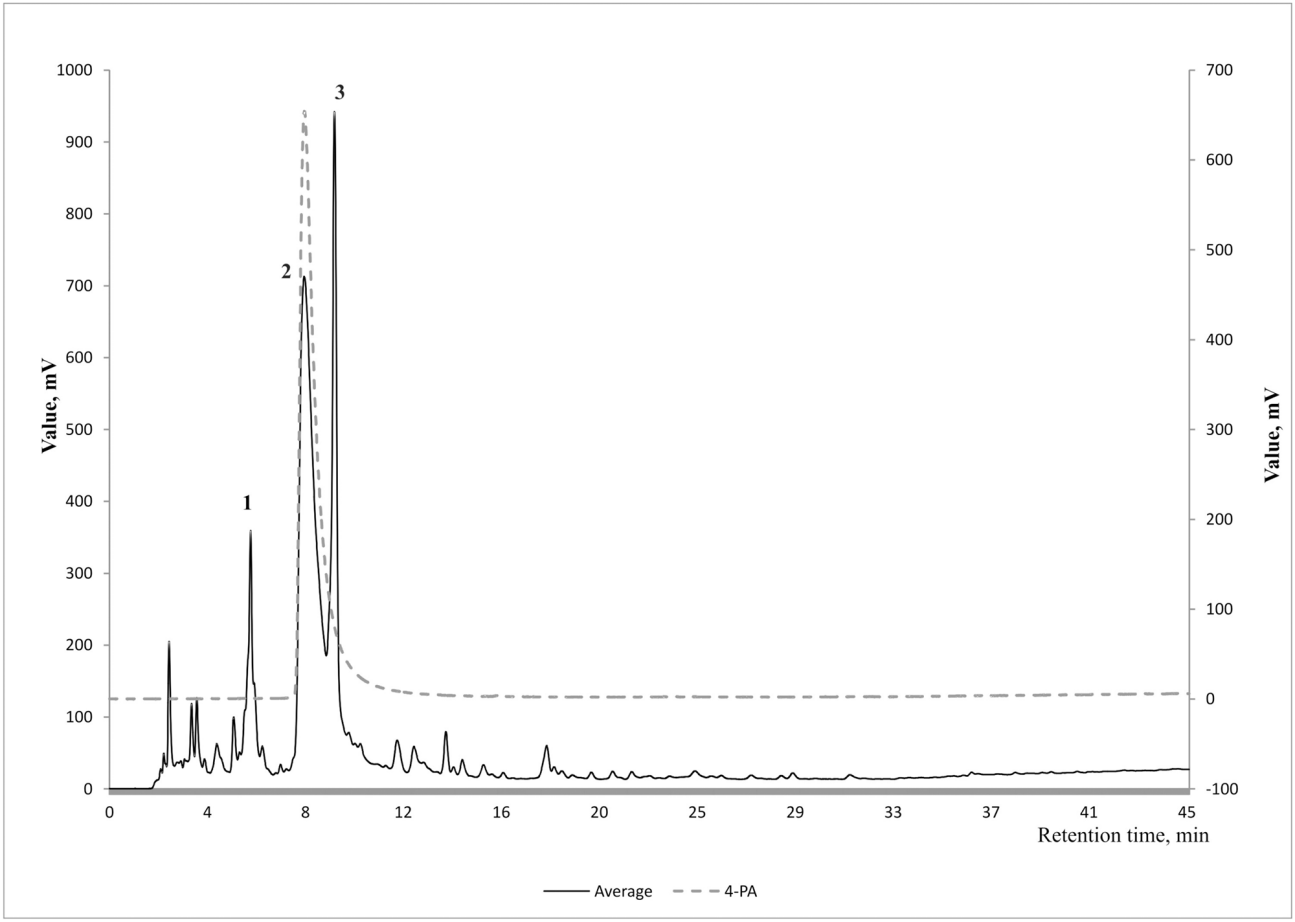
Example of an averaged Ex320/Em430 nm chromatogram of the spent dialysate (N = 10). Samples were collected 7–10 min after the start of the dialysis. Compound 2 was identified as 4-pyridoxic acid. Raised chromatogram is of a reference 4-pyridoxic acid solution (1 μM).

Fig 2 illustrates an example of averaged HPLC fluorescence chromatograms (Ex320/Em430 nm) of spent dialysate of 9 different patients’ collected 7–10 minutes after the start of HD sessions (for specific data see Figure B in S1 File). The peak number 2 was found to coincide with 4-PA on the basis of retention time and mass spectrum.

### 3.2. Identification of 4-PA

**Fig 3:**
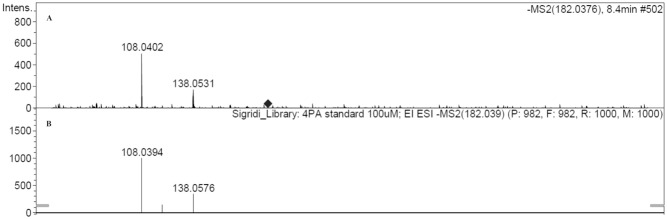
Comparison of mass spectra (negative ionization) of A) biggest fluorescent peak (no 2, Fig 2) and B) 4-pyridoxic acid reference.

The MS spectrum of the highest peak no 2 (Fig 3A) was compared to the spectrum of the reference substance (Fig 3B) where a good match with 4-PA was found.

### 3.3. Contribution and concentration of 4-PA

All 39 dialysis sessions followed were used to calculate concentration of 4-PA found chromatographically in spent dialysates. It was found that the average concentration of 4-PA in the beginning of dialysis was 4.20 ± 2.29 μmol/L and in the end 1.71 ± 0.67 μmol/L. Average contribution of fluorophores in HPLC fluorescence chromatograms of the spent dialysates were calculated using 10 dialysis sessions data. The calculation showed that 4-PA appears to be the main contributor of the fluorescence signal (Table 2) at Ex320/Em430 nm (for the dataset see Table A in S1 File).

**Table 2:** Mean contribution (Mean ± SD, N = 10) values in percentage for main fluorescent peaks in the spent dialysate samples (Ex320/Em430 nm).

	Mean contribution ± SD at the start of the dialysis	Mean contribution ± SD at the end of the dialysis
Unknown 1	7.4 ± 2.1	7.2 ± 3.1
4- PA (peak 2)	42.2 ± 17.0	47.7 ± 18.0
Unknown 3	22.4 ± 5.4	19.9 ± 3.1

### 3.4. Linear correlation

**Fig 4:**
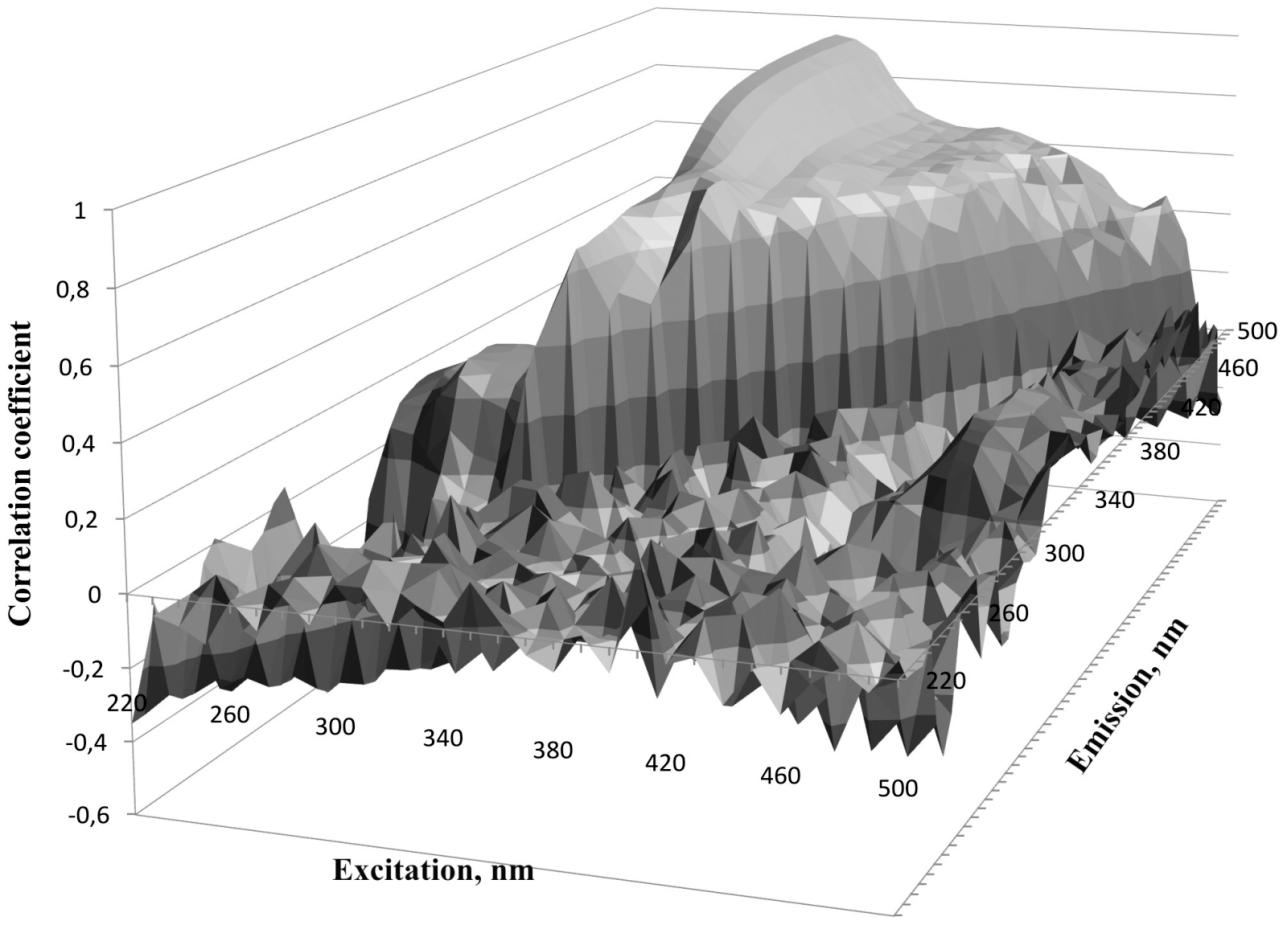
Dependence of the correlation between fluorescence intensity of spent dialysate and 4-pyridoxic acid concentration on the excitation-emission wavelength conditions.

Linear correlation was calculated between concentration of 4-PA found chromatographically in spent dialysate and directly measured fluorescence intensity of the dialysate. High correlation (R > 0.88, N = 195) was found in the wavelength region Ex310-330/Em415-500 nm (Fig 4). For correlation on a wider scale of Ex220-500/Em220-500 nm see Figure C in S1 File.

**Fig 5:**
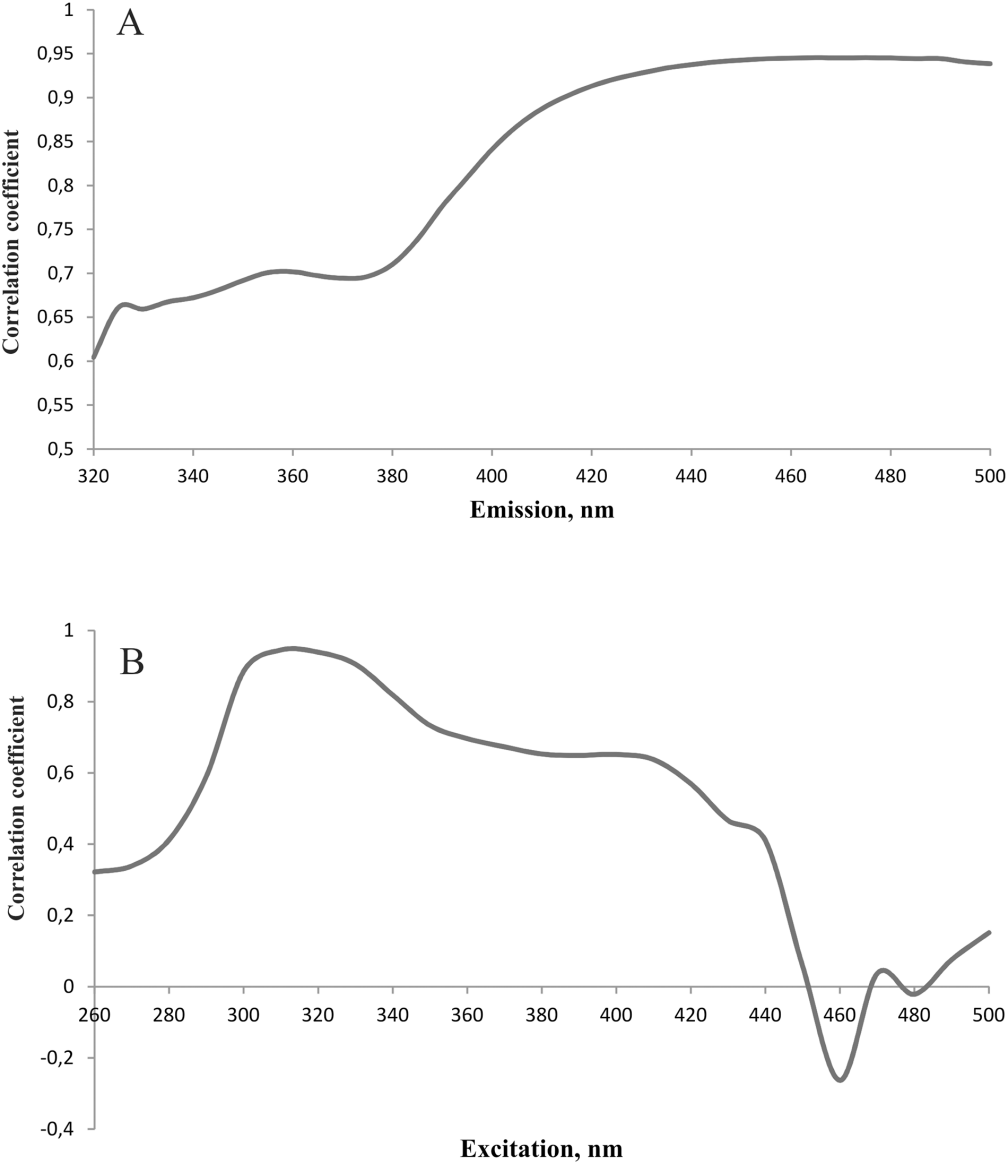
Correlation between fluorescence intensity of spent dialysate and 4-pyridoxic acid concentration at Ex310 nm (A) and at Em460 (B). The best correlation was found with Ex310/Em460 nm (R_max_ value of 0.95, N = 195).

More detailed examination of the region with the highest R values revealed that the best correlation was found at the wavelengths Ex310/Em460 nm (R_max_ = 0.95, N = 195 (Fig 5, for specific data see Figure D in S1 File).

**Fig 6:**
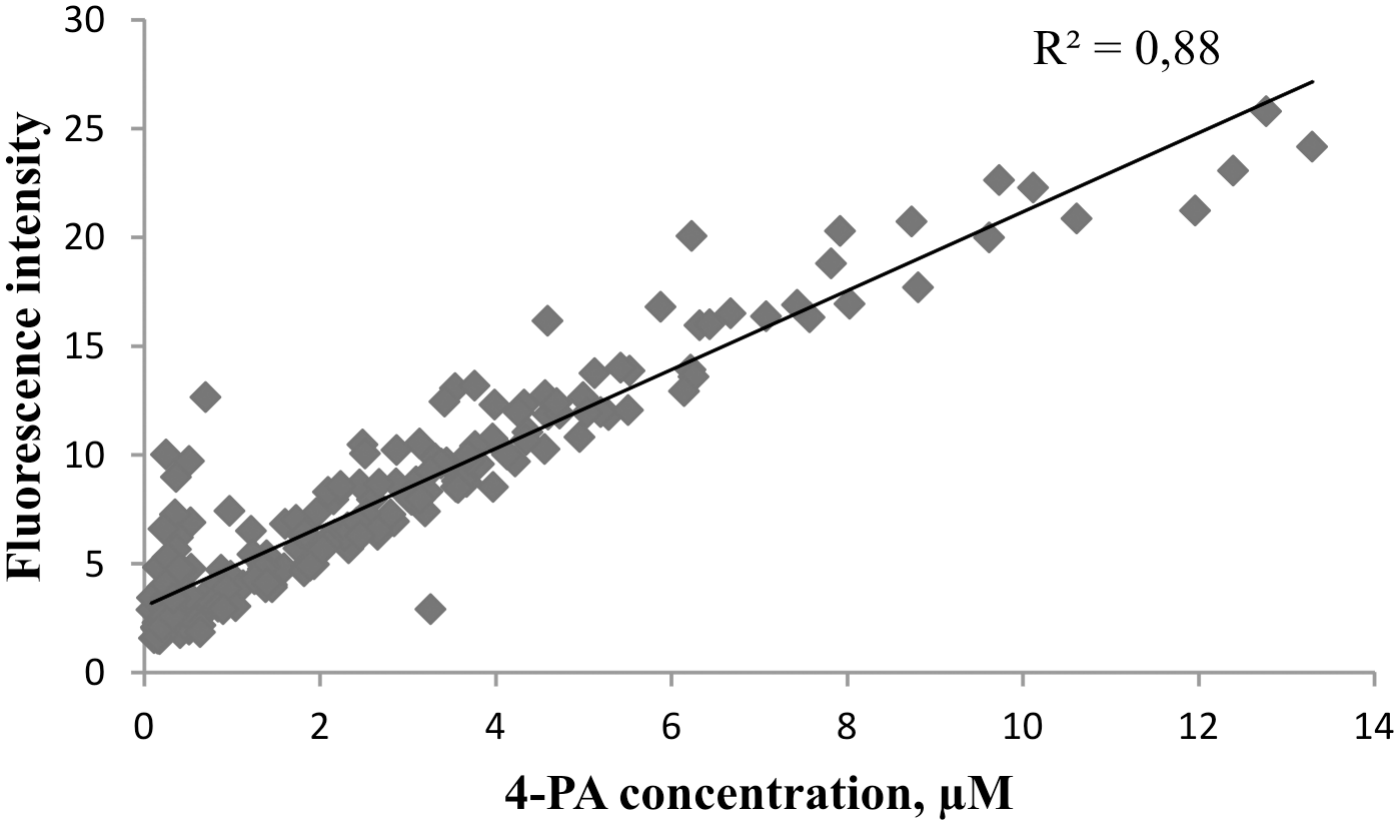
An example of the regression line between concentration of 4-PA in spent dialysate and fluorescence intensity (Ex310/Em460 nm, *p* <0.0001, N = 195).

Fig 6 presents the regression equation of 4-PA against fluorescence intensity at wavelengths of highest correlation Ex310/Em460 nm in the spent dialysate (for specific data see Figure E in S1 File).

## Discussion

As vitamin B6 is commonly used in the treatment of patients of chronic kidney disease, the monitoring of the vitamin status of the patient is important. Our present study confirms that measurement of fluorescence in spent dialysate may be useful for assessment of elimination of 4PA, as the main metabolite of B6, from the blood of dialysis patients receiving regular B6 treatment.

PLP, the main active form of B6 is mostly used to evaluate the status of B6. However plasma PLP levels are low in dialysis patients [21,25] and thus laborious to measure. As plasma 4-PA concentrations are elevated in renal disease it might be used as an indicator of renal function [22]. Moreover 4-PA is not protein bound unlike most of PLP and pyridoxal [26] thus making measurements easier. Another potential benefit of monitoring 4-PA could be the correlation between 4-PA and other compounds like creatinine and urea [27], plasma PLP and homocysteine [21,28–30], whereas higher homocysteine levels are a risk factor for vascular disease [19,22]. Correlation between above mentioned compounds and plasma 4-PA as well as advanced glycation end product pentosidine [21] makes 4-PA potentially a versatile marker for the assessment of multiple aspects of health status of the dialysis patient and not only the B6 catabolism. We studied the possibility to monitor the elimination of 4-PA from the blood of a dialysis patient by fluorescence measurements in the spent dialysate.

To our knowledge, B6 catabolite 4-PA has not been measured in spent dialysate so far. Many 4-PA measurement methods consist of several derivatization steps to enhance fluorescence intensity [19,26]. Cabo *et al*. [26] have done an extensive review on different measuring methods where 4-PA concentration can be measured in down to nanomolar quantities. The drawback of those methods is that they have many preparation steps and use toxic reagents. The results of this study show that 4-PA concentrations in spent dialysate of ESRD patients are high enough (micromolar range) (Fig 6) for direct fluorescence measurements. The pyridoxic acid has been found in different forms in human plasma and urine, as 4-PA and 4/5-pyridoxolactone [31] with different fluorescent characteristics [32]. Contrariwise to these observations we could not find pyridoxolactone on our MS chromatograms of dialysate. Therefore, we cannot connect comparatively long wavelength interval of correlation between emission and 4-PA content in dialysate (Fig 5A) with possibility of lactonization of the 4-PA. Some lactonization has been found to take place also in the time of laboratory manipulations of plasma samples [21]. Correlation between 4-PA and pentosidine content has been found in plasma [21]; it cannot be excluded that some AGE compound with emission up to 460 nm may be involved in dialysate emission together with 4-PA in dialysate also. Nevertheless quite good correlation between 4-PA concentration and fluorescence of spent dialysate (Figs 4–6) was obtained.

The results of this study indicate that the majority of the fluorescence signal (Ex320/Em430 nm) derives from 4-PA, which is the biggest contributor to the fluorescence signal with 42.2±17.0% to the total fluorescence intensity at the beginning of the dialysis and 47.7±18.0% at the end (Table 2, Figs 2 and 3). Also it has to been taken into account that there is inter- and intraindividual variation of 4-PA concentration and fluorescence intensity in the spent dialysate. The most suitable fluorescence regions for 4-PA assessment with sufficiently high correlation (R>0.88) appear to be in the wavelength region Ex310-330/Em415-500 nm (Figs 4 and 5) with the highest correlation (R_max_ = 0.95, *p*<0.0001) at Ex310/Em460 nm. The maximum correlation wavelength may be different from the 4-PA maximum wavelength of Ex320/Em425nm (Fig 1) due to the different proportion of fluorophores and their change during dialysis.

As 4-PA may be a potentially versatile marker that may help assess multiple aspects of health status of the dialysis patient an easy measurement method would be beneficial for the patient and hospital staff. Our previous study showed that spent dialysate provides a good substitution for blood for diagnostic analyses [33]. Sampling is easier, it does not disturb the patient and the analysis is simpler. This study suggests that 4-PA can be followed by simple direct fluorescence measurement also and provide hospital staff an opportunity for real-time monitoring of 4-PA elimination of the ESRD patient currently treated as well as follow the tendency of changing of the status during long-time dialysis treatment of the particulate patient.

Consequently, on-line fluorescence measurements in the region of Ex310-330/Em415-500 nm could potentially help for assessment of the status of the vitamin B-6 metabolism of ESRD patients during regular dialysis treatment.

The limitations of this study were relatively small data material (only 10 dialysis patients during 40 dialysis sessions were included) and the study did not cover heterogeneity of total dialysis population. The possible role of AGE-s in 4-PA-linked fluorescence as well as the possibility of not-radiative energy transfer (FRET) between fluorophores in dialysate need to be explained for final interpretation of the wavelength shift on the correlation graph (Fig 5A) compared to emission maximum of the 4-PA itself.

## Conclusion

The 4-pyridox acid (4-PA) appeared to be the highest contributing peak of the HPLC chromatogram measured in the wavelength of Ex320/Em430 nm. The intensity of the fluorescence in the region Ex310-330/Em415-500 nm has high (R>0.88) correlation with 4-PA concentration in spent dialysate. It can be concluded from these observations, that 4-PA elimination from the blood of end stage renal disease patients can be potentially followed using monitoring of the fluorescence of the spent dialysate during regular dialysis treatment.

## Supporting Information

S1 File Includes the following: Figure A: data for normalized emission spectra of spent dialysate and 4-pyridoxic acid at excitation of 320 nm. Figure B: data for an averaged Ex320/Em430 nm chromatogram of the spent dialysate. Table A: data for mean contribution calculations. Figure C: dependence of the correlation between fluorescence intensity of spent dialysate and 4-pyridoxic acid concentration. Figure D: Data for the correlation between fluorescence intensity of spent dialysate and 4-pyridoxic acid concentration at Ex310 nm and at Em460. Figure E: Data of an example of the regression line between concentration of 4-PA in spent dialysate and fluorescence intensity.
(XLSX)
